# Environmentally Friendly Wheat Farming: Biological and Economic Efficiency of Three Treatments to Control Fungal Diseases in Winter Wheat (*Triticum aestivum* L.) under Field Conditions

**DOI:** 10.3390/plants11121566

**Published:** 2022-06-14

**Authors:** Nazih Y. Rebouh, Toufik Aliat, Petr M. Polityko, Dalila Kherchouche, Nadia Boulelouah, Sulukhan K. Temirbekova, Yuliya V. Afanasyeva, Dmitry E. Kucher, Vadim G. Plushikov, Elena A. Parakhina, Mourad Latati, Anvar S. Gadzhikurbanov

**Affiliations:** 1 Department of Environmental Management, Peoples’ Friendship University of Russia (RUDN University), 117198 Moscow, Russia; kucher-de@rudn.ru (D.E.K.); pliushchikov-vg@rudn.ru (V.G.P.); parakhina-ea@rudn.ru (E.A.P.); m.latati@yahoo.com (M.L.); 2 Forest Management Department, Higher National School of Forests, Khenchela 40000, Algeria; toufikaliatensf2021@gmail.com; 3 GNU Moscow Research Institute of Agriculture “Nemchinovka”, 143026 Moscow, Russia; polityko_petr@mail.ru; 4 Department of Agronomy, Institute of Veterinary and Agronomy Sciences, University Batna 1, Batna 05000, Algeria; dalila.kherchouche@univ-batna.dz (D.K.); boulelouahnadia@gmail.com (N.B.); 5 All-Russian Research Institute of Phytopathology, Bolshye Vyazyomy, Odintsovo District, 143050 Moscow, Russia; sul20@yandex.ru; 6 Federal Horticultural Center for Breeding, Agrotechnology and Nursery, 115598 Moscow, Russia; yuliya_afanaseva_90@bk.ru; 7 Laboratoire d’Amélioration Intégrative des Productions Végétales (AIPV: C2711100), Ecole Nationale Supérieure Agronomique de Productions Végétales, Algiers 16200, Algeria; 8 Federal Research Centre “Fundamentals of Biotechnology” of the Russian Academy of Sciences, 119071 Moscow, Russia; gadcgikurbanow@mail.ru

**Keywords:** biopesticide, biotic stress, conservation agriculture, farm economics, fungal pathogens

## Abstract

The control of wheat diseases using bioagents is not well studied under field conditions. The present study was aimed at investigating, during four consecutive growing seasons (2017–2020), the efficacy of two integrated crop protection (ICP) systems to control the common wheat diseases for enhancing the productivity and profitability of winter wheat crops and ensuring nutritional and food security. Two environmental-friendly treatments were tested, biological (T1), which contained bioagents and fertilizers, and combined (T2), which included fertilizers and bioagents coupled with lower doses of fungicides. The chemical treatment (T3) was used for comparison with (T1) and (T2). Furthermore, two Russian winter wheat varieties (Nemchinovskaya 17 (V1) and Moscovskaya 40 (V2)) were studied. A randomized complete block design was used with four replicates. Diseases infestation rates for snow mold (SM), root rot (RR), powdery mildew (PM), and *Fusarium* (Fus), yield performances, and grain quality (measured through protein content) were determined according to the tested treatments, and the economic efficiency was calculated for each treatment. The combined treatment (T2) was the most effective against fungal diseases with 1.8% (SM), 1.2% (RR), 0.9% (PM), and 0.9% (Fus). The highest grain yield (6.8 t·ha^−1^), protein content (15.2%), and 1000-grain weight (43.7%) were observed for winter wheat variety Moscovskaya 40 with the combined treatment (T2). The highest number of productive stems (N.P.S) (556 stems/m^2^) was attained for combined treatment (T2), followed by biological treatment (T1) (552 stems/m^2^) with the variety Nemchinovskaya 17. The profitability (cost–benefit ratio) of the combined treatment (T2) was 2.38 with the Moscovskaya 40 variety (V2), while 2.03 was recorded for the biological treatment. Applying environmentally friendly combined and biological treatments resulted in high wheat yield and net income, as well as healthy products.

## 1. Introduction

Wheat is among the staple cereal crops worldwide, providing nearly 55% of the carbohydrates and daily proteins for 85% of the world’s population [1,2]. This crop is directly related to food security and the global economy [3]. However, root rot caused by *Fusarium culmorum* and *Fusarium avenaceum,* head blight (FHB) caused by *Fusarium graminearum*, snow mold caused by *Microdochium nivale* and *Fusarium nival*, and powdery mildew caused by *Blumeria graminis* are the most widespread and harmful wheat diseases worldwide [4,5,6]. Consequently, these fungal diseases can cause colossal yield losses of up to 20% of total wheat production if an efficient plant protection system is not established [7].

For environmental and economic reasons, wheat cultivation technology needs improvements [8,9]. The adoption of new environmentally friendly practices such as integrated wheat crop protection systems has become necessary to reduce yield losses caused by biotic stresses, as well as obtain healthier products that meet the dietary needs of a growing population, given that the demand for organic foods has grown rapidly over the past three decades [10].

An integrated crop protection system (ICPS) can be established taking into account the other agricultural practices [11], namely, the fertilization system, which is in a close relationship with the protection system [12], the crop rotation system, used as a natural barrier against a wide range of soil-borne fungal pathogens, the intercropping system, and plant breeding by developing new cultivars adapted both to the specific climatic conditions of the regions and to biotic stresses [13,14,15]. For example, according to Flower et al. (2019) [16], fallow and lupin in the farmer rotation system are the most effective at reducing pathogens levels. Furthermore, Campanella et al. (2020) [17] reported that the use of *Brassica carinata* as a precursor crop significantly reduced root rot infestation in wheat due to the production of volatile sulfur compounds and changes in soil microbial community composition. El-Mehy et al. 2022 [18] demonstrated that intercropping with garlic significantly reduced the disease infestation rate of root rot and damping-off diseases. Similarly, Gomez-Rodriguez et al. (2007) [19] found that intercropping tomato–marigold significantly reduced early blight caused by *Alternaria solani*. Moreover, through a selection program, Temirbekova et al. (2021) [20] created outstanding wheat varieties resistant to several types of fungal disease such as *Septoria* head and leaf blotch, *Fusarium* head blight, root rot, and yellow dwarf virus. In addition, several wheat breeding programs have developed resistance genes to powdery mildew, Karnal bunt, and many other diseases [21,22], giving cultivars a key role in establishing an integrated crop protection system.

Biopesticides, using biological agents to control disease, such as *Pseudomonas* spp., *Bacillus* spp., *Burkholderia* spp., and *Trichoderma* spp., are considered within the framework of integrated plant protection strategies [23,24,25]. Nevertheless, the use of only biopesticides is not ample to obtain higher crop productivity due to the complexity of the rhizosphere and the need to apply a high amount of these bioagents to cover the whole rhizosphere [26]. Lahlali et al. (2022) [27] reported that the use of bioagents in crop protection systems is not always efficient to control plant pathogens. The authors explained their approach by the presence of several factors affecting the success of biological control against plant pathogens, such as the lack of studies under field conditions and the resistance of fungal pathogens to biofungicides. In this regard, the use of chemical control has increased so as to achieve relatively stable yields [28], which subsequently increases environmental pollution and accumulates chemical residues in the agroecosystem.

Environmentally friendly farming strategies include the combination of some practices in order to benefit from the interactions between them that ensure obtaining high yields while respecting the environment [29]. However, the use of bioagents in combination with compatible pesticides and the interactions of this combination with other agricultural practices are poorly studied and need to be improved, especially under field conditions. In this context, the current study was aimed at (1) investigating the effect of combined treatment including biological components coupled with a lower dosage of fungicides to control wheat diseases under field conditions, (2) comparing the effect of biological treatment with that of chemical treatment, and (3) determining the most productive and profitable variety–treatment combination for wheat cultivation in the Central Non-Black Earth region of Russia.

## 2. Results

### 2.1. Effect of the Tested Treatments on Diseases Infestation

**Figure 1:**
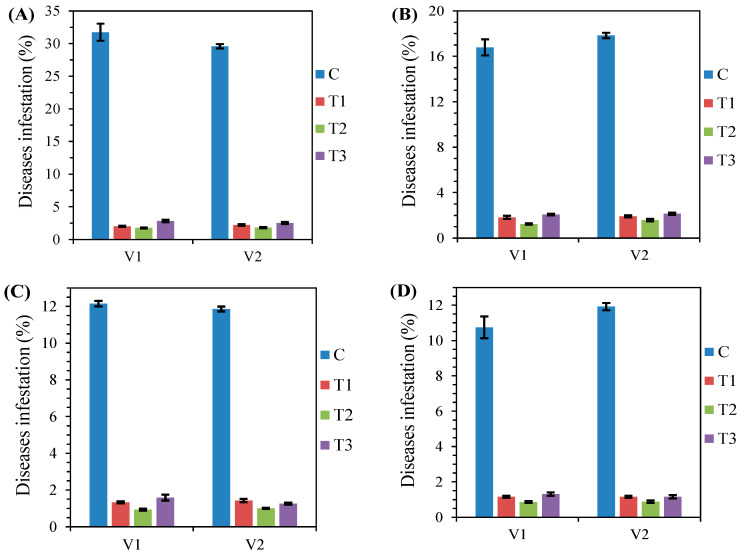
Bar charts showing disease rates including snow mold (**A**), root rot (**B**), powdery mildew (**C**), and *Fusarium* (**D**) in the two studied varieties (Nemchinovskaya 17 variety (V1) and Moscovskaya 40 variety (V2)) according to the tested treatments (T1—biological, T2—combined, and T3—chemical) (2017–2020).

Figure 1 shows that the three tested treatments considerably reduced the infestation rate of the four studied diseases. For example, the rate of snow mold (SM) disease fell from 31% (control) to less than 3% (all treatments). Whatever the disease, the lowest rate was obtained by combined treatment (T2), with values varying from 0.86% for *Fusarium* recorded by Nemchinovskaya 17 variety (V1) (Figure 1D) to 1.82% for snow mold registered by Moscovskaya 40 variety (V2) (Figure 1A).

**Figure 2:**
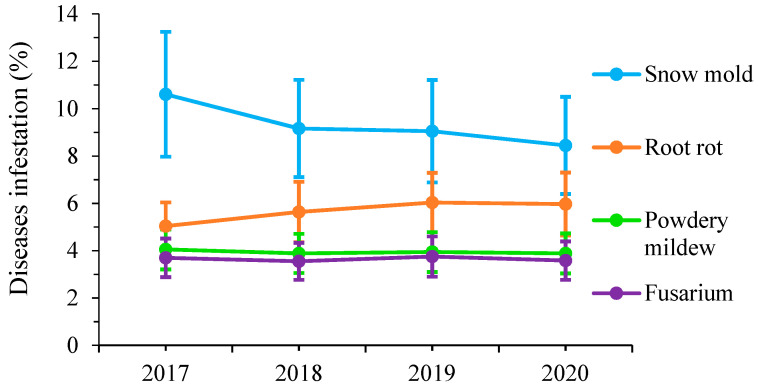
Disease rate dynamics (2017–2020).

The rate of disease dynamics according to the years is represented in the Figure 2. Powdery mildew (PM) and *Fusarium* (Fus) diseases showed a stable rate from 2017 to 2020, not exceeding the value of 4%. For snow mold (SM) and root rot (RR), disease rate dynamics were reversed; it tended to decrease for snow mold (SM), from 10.61% in 2017 to 8.45% in 2020, while it tended to increase slightly for root rot (RR), from 5.04% in 2017 to 5.97% in 2020.

Treatments and varieties tested had a significant effect on disease rate in all the studied varieties (*p* ≤ 0.001), except for powdery mildew (PM) and *Fusarium* rates, for which the varieties did not have a significant effect. However, the analysis carried out revealed that the interaction (variety × treatment) had a significant effect on the snow mold (SM), root rot (RR), powdery mildew (PM), and *Fusarium* (Fus) rates with *p* ≤ 0.001, *p* ≤ 0.001, *p* ≤ 0.01, and *p* ≤ 0.05, respectively (Table 1).

### 2.2. Effect of the Tested Treatments on the Wheat Yield and Grain Quality

**Figure 3:**
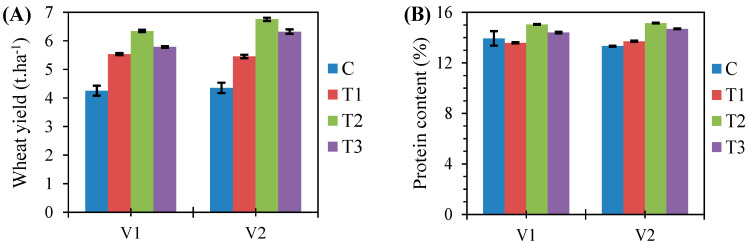
Bar charts showing wheat yield (**A**) and protein rate (**B**) in the two studied varieties (Nemchinovskaya 17 variety (V1) and Moscovskaya 40 variety (V2)) according to the tested treatments (T1—biological, T2—combined, and T3—chemical) (2017–2020).

Figure 3A displays the yield performances of the two winter wheat varieties attributed to the treatments. Combined treatment (T2) gave the best yields whatever the considered wheat variety: 6.3 ± 0.04 t·ha^−1^ and 6.8 ± 0.05 t·ha^−1^ for the Nemchinovckaya 17 variety (V1) and Moscovckaya 40 variety (V2), respectively. The increase in yield was 2 t·ha^−1^ compared to the control (C). On the other hand, the lowest yield was recorded when biological treatment was implemented (T1) in all wheat varieties: 5.5 ± 0.06 (V1) and 5.5 ± 0.04 t·ha^−1^ (V2). According to combined treatment (T2), comparable values of protein content were recorded for both varieties: 15.05% and 15.16% with the Nemchinovckaya 17 variety (V1) and Moscovckaya 40 variety (V2), respectively (Figure 3B).

In Table 2, the analysis of variance for grain yield and quality (protein content) revealed that the treatment, variety, and their interaction (treatment × variety) had a highly significant effect on the grain yield (*p* ≤ 0.01). However, the grain quality was significantly affected only by the studied treatments (*p* ≤ 0.001).

The results showed that the tested treatments had a highly significant effect on the yield components expressed by the number of productive stems (NPS) and 1000-grain weight (*p* ≤ 0.001). It is shown that variety affected and strengthened the yield structure since the *p*-value was ≤0.001. In addition, the interaction (treatment × variety) had a significant effect on the number of productive stems (NPS) and 1000-grain weight (*p* ≤ 0.001 and *p* ≤ 0.05, respectively) (Table 3).

### 2.3. Principal Component Analysis

**Figure 4:**
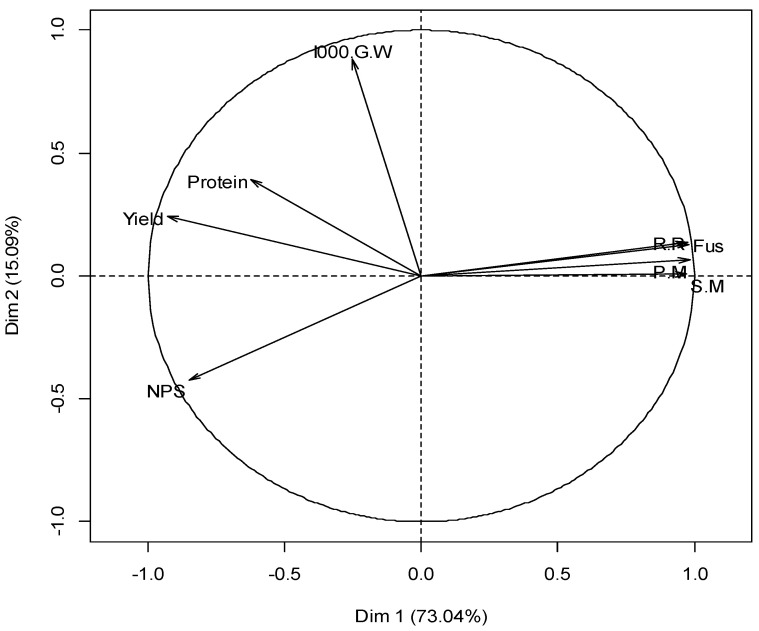
Trend and relationship between the distribution of agronomic parameters and the rates of the studied diseases.

Figure 4 illustrates the results of a principal component analysis (PCA) developed for the different agronomic parameters studied to characterize the treatment × variety × year interactions. The circle indicates the projection of the various variables studied along the horizontal axis (axis 1) and the vertical axis (axis 2) = DIM1 and DIM2.

– The percentage of the variation explained by the first two axes was 88.13% (axis 1 = 73.04% and axis 2 = 15.09%). This provides information on the agronomic parameter distribution and the rates of the four studied diseases. These parameters were evenly distributed on the two axes (DIM 1 and DIM 2).
– The yield and NPS parameters strongly and negatively correlated with axis 1 compared to the protein content, which presented a moderate correlation. Conversely to these parameters, the four variables related to the different diseases (snow mold, root rot, powdery mildew, and *Fusarium*) were very strongly and positively correlated with the same axis.
– The 1000-grain weight (1000 GW) variable was the only variable positively correlated with axis 2.

### 2.4. Projection of Individuals

**Figure 5:**
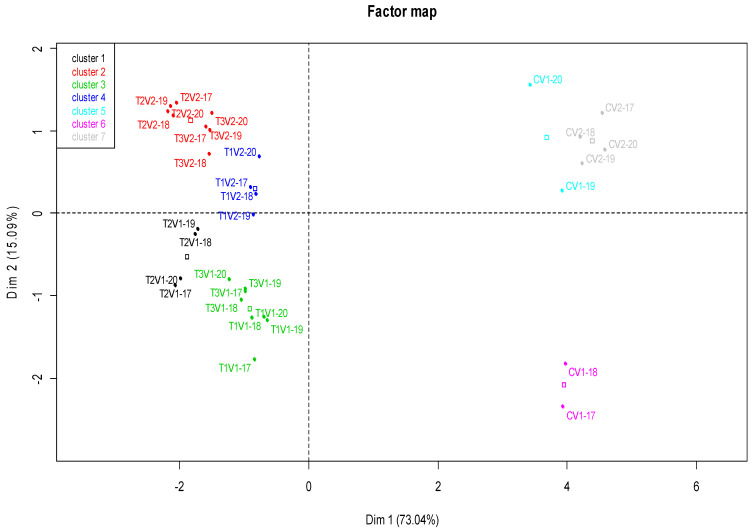
Projection of individuals in the 1 × 2 factorial plan.

Figure 5 illustrates the projection of individuals (treatment × variety × year interactions) on plans 1 and 2 divided into two large opposing clusters. The first large group, located to the left of the plan, was made up of the interactions of the three treatments with the two varieties (T1V1, T1V2, T2V1, T2V2, T3V1, and T3V2) characterized by better yield and grain quality, as well as a low disease rate. Conversely, the second large group, located on the right, included the control × variety interactions (CV1 and CV2) characterized by a high disease rate, low yield, and poor grain quality. Two individuals CV1-17 and CV1-18 were separated from the large group by their high NPS and low 1000-grain weight.

The group formed by the T2V2 and T3V2 interactions was seemingly the best ranked, characterized by a better quantitative and qualitative yield with a high 1000-grain weight and better resistance to the studied diseases during all years. However, the four individuals of the T2V2 interaction were considered better than those of the T3V2 interaction.

### 2.5. Relationships among the Studied Variables

**Figure 6:**
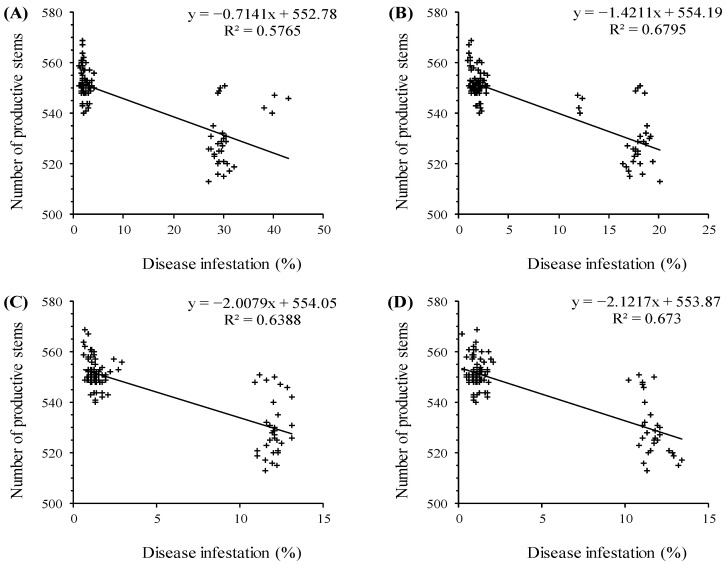
The relationship between “number of productive stems” and “disease infestation”: snow mold (**A**), root rot (**B**), powdery mildew (**C**), and *Fusarium* (**D**).

Figure 6 shows the relationship between NPS and disease infestation rate using the linear regression method. The disease infestation rate was negatively correlated with the number of productive stems (*r*^2^ = 0.57, *r*^2^ = 0.67, *r*^2^ = 0.63, and *r*^2^ = 0.67 for snow mold, root rot, powdery mildew, and *Fusarium*, respectively) (Figure 6A–D). Specifically, the number of productive stems decreased with increasing disease infestation rate.

**Figure 7:**
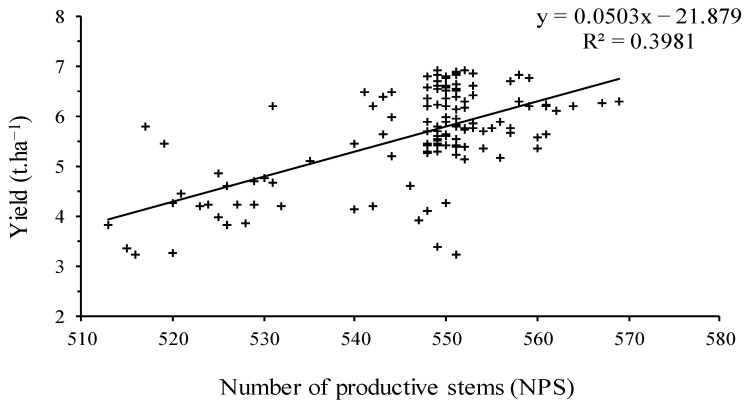
The relationship between “yield” and “number of productive stems”.

The relationship between the number of productive stems and yield is shown in Figure 7 using linear regression. The number of productive stems was moderately correlated with grain yield (*r*^2^ = 0.39). Specifically, the grain yield increased with an increase in the number of productive stems.

### 2.6. Economic Efficiency of Tested Treatments

The economic efficiency (EE) of cropping systems is very important when setting up cultivation strategies. It is the degree or ability of a farmer to produce a given level of output at the least cost. Table 4 illustrates the economic efficiency of two studied winter wheat varieties affected by the applied treatments. The highest income was achieved with the combined treatment (T2) in the Moscovskaya 40 variety (V2) when net income was 62.300 RUB·ha^−1^. The most profitable treatment was chemical treatment (T3) tested with the Moscovskaya 40 variety (V2) with the highest payback (2.65 RUB), followed by the combined treatment (T2) with the same variety (2.38 RUB). However, the lowest profitability (cost–benefit ratio of 2.03) was observed when the biological treatment was applied to all studied varieties.

## 3. Discussion

In the present research, the influence of two environmental friendly crop protection systems on the disease incidence, yield, and grain quality of winter wheat was investigated and compared to the chemical system.

The results showed that the combined treatment (T2) significantly decreased the snow mold infestation rate in the two winter wheat varieties studied. Snow mold infestation was reduced by 94% compared to the control (C) (Figure 1A). This was probably due to the pre-sowing seed treatment carried out by *Bacillus subtilis* combined with the lower dose of flutriafol, thiabendazole, and imazalil, since *B. subtilis* promotes germination and plant growth, which enhances plant resistance to root disease [30]. In addition, our previous investigation [11] demonstrated that flutriafol, thiabendazole, and imazalil molecules significantly decreased snow mold infestation in winter wheat, in line with the current study. However, no previous studies investigated the effect of combined bioagents/fungicides to control wheat snow mold disease.

The harmful wheat root rot disease was significantly reduced through the combined (T2) and biological (T1) treatments that demonstrated a high ability to decrease it. This may be because of the several possible mechanisms involved in biological control by *B. subtilis* and *B. megaterium* leading to the production of antifungal substances, as explained by Ryder et al. (1998) [31]. Our results agree with those reported by Marzouk et al. (2021) [32]. The authors showed that *B. subtilis* and *B. megaterium* were selected as the most effective stains to control root rot disease among several microorganisms studied. Furthermore, other studies noted that seed treatments with systemic fungicides such as thiabendazole and imazalil significantly reduced root rot disease abundance [33,34]. On another note, in line with the obtained results in our study, Omar et al. (2006) [35] reported that thiabendazole, which belongs to the benzimidazole family, led to a reduction in root rot disease symptoms by 84%. Moreover, a lower infestation rate was recorded with all studied treatments in this experiment. This can be best explained by the tillage depth adopted, which was 22 cm. At this depth, crop residues are buried deeply in the soil, which accelerates their degradation, thus limiting the inoculum of phytopathogens [36].

Powdery mildew is one of the most devastating diseases of common wheat. It can be virtually found wherever wheat is grown at varying degrees [37]. For the two studied winter wheat varieties, the lowest powdery mildew infestation rate was achieved by the combined treatment (T2), where *B. subtilis*, *Pseudomonas fluorescens*, and *Trichoderma harzianumat* were applied in combination with flutriafol and tebuconazole at the tillering and elongation of stem phases. These results are similar to those reported by Ahmed et al. (2021) [38], who found that *B. subtilis, B. megaterium, P. fluorescens*, and *T. harzianum* expressed high efficiency in suppressing powdery mildew. Similarly, Gilardi et al. (2008) [39] mentioned that the foliar spraying by *B. subtilis* with azoxystrobin allowed effective disease control against powdery mildew. Indeed, in our field experiment, the fungicide CONSUL (flutriafol + azoxystrobine) and the biofungicide GAMAIR (*B. subtilis*) were applied at the earing stage, which represents the most delicate phase of wheat. In this earing stage, infected kernels are poorly developed, small, and sometimes deformed, which can significantly reduce grain yield [40].

The combined treatment (T2) was the most efficient method adopted to control *Fusarium*, probably due to the fungicide used (tebuconazole molecule). The tebuconazole molecule invariably showed high efficiency in limiting the disease level severity [41], especially in *Fusarium* spp. Additionally, studies revealed that this fungus is more susceptible to triazoles and tebuconazole [42]. Furthermore, Nourozian et al. (2006) [43] found that *B. subtilis, P. fluorescens*, and *Streptomyces* spp. were potential biological agents for the control of *Fusarium* head blight (FHB). Guimaraes et al. (2020) [44] evaluated the use of bioagents (*B. subtilis + Streptomyces araujoniae*), in combination with fungicides (cyproconazole + azoxystrobin), on the incidence of *Fusarium verticillioides* in maize crop. These authors found that spraying bioagents separately from fungicides was less efficient in controlling diseases than combination spraying. The results are in close agreement with those reported in the present study, in which only chemical and biological treatments showed a lower efficiency compared to the combined way.

The compatibility of bioagents with fungicide is an essential factor when selecting molecules and microorganisms to set up a combined crop protection system. Fungicides may have detrimental effects on both the pathogen and the antagonist. There are several studies which analyzed the compatibility of fungicides with bioagents. For example, Rajkumar et al. (2018) [45] reported that carbendazim (benzimidazole) is more compatible with *B. subtilis* compared to other studied fungicides. Ahila Devi et al. (2020) [46] mentioned that *P. fluorescens* and *B. subtilis* had high compatibility with azoxystrobin. In the same context, Sameer (2019) [47] concluded that the efficiency of *T. harzianum* and *B. subtilis* in controlling root rot disease was enhanced by its compatibility with low rates of the tested fungicides when investigating the compatibility of biological control agents with fungicides against root rot diseases of wheat. These results are in line with those obtained in our study and confirm the choice of the combinations for which we opted.

The timing and frequency of fertilizer application are essential in integrated wheat management and may improve the plant protection system efficiency. In the present experiment, all treatments demonstrated high efficiency in controlling snow mold disease. This is probably due to the top dressing by nitrogen (N) carried out at the tillering stage of wheat. In this growth stage, the plants become weakened due to the long period spent under the snow cover, which subjects them to an easy target for different infections, mainly cryophilic fungi, as demonstrated by Temirbekova et al. (2022) [48]. Moreover, the form of applied nitrogen may play a crucial role in disease management [49]. It has been concluded in many studies that the use of ammonia (NH_4_-N) increased the disease severity of *F. culmorium*, contrary to nitrate (NO_3_-N) which decreased it [50,51,52]. In our study, nitrate (NO_3_-N) was applied using different studied treatments, which explains the lowest infestation rate of root rot disease. Ultimately, nutrients may decrease disease infestation to an acceptable level or at least to a level at which other cultural practices will be more successful and less expensive [53].

The natural life cycle of soil and airborne pathogens is broken when the crop rotation system is carried out [54,55]. In our field experiment, the precursor was pea crop, which led to a decrease in root rot disease infestation, probably due to reduced inoculum density in soil and changes in soil microbe populations. Similarly, Woo et al. (2020) [56] reported that pea–wheat rotation reduces the abundance of *F. graminearum*. Furthermore, Borrell et al. (2017) [57] noted that pea, lentil, and chickpea used in crop rotation influence the fungal community of wheat, promoting the activity of arbuscular mycorrhizal fungi associated with wheat. In addition, Mc Key and Reader (1953) [58] noted that less snow mold occurs on wheat when using alfalfa, sweet clover, or pea crops as the precursor, contrary to the cultivation of wheat in a monoculture, where the severity of the disease increases.

The genetic resistance of the cultivars plays a primordial role when setting up an integrated crop protection system [59]. High integrated disease control can be achieved by synergy, involving the sustainable improvement of all agricultural practices to benefit from the interactions between them and promoting the genetic potential of resistant cultivars. In the present study, the used varieties (Nemchinovckaya 17 variety (V1) and Moscovckaya 40 variety (V2) are among the most resistant varieties in Russia [60]. Petrov et al. (2016) [61] reported that the Nemchinovckaya 17 variety and Moscovckaya 40 variety were the most resistant varieties to snow mold and powdery mildew when investigating the resistance of 10 varieties to wheat diseases in the Nizhniy Novgorod region. Our results are in line with those reported by these authors and revealed the intrinsic genetic performance of the studied varieties for resistance to several diseases.

Crop yield is the determinate factor in the selection of a specific treatment. The current study revealed that the combined treatment (T2) increased wheat yield by 2 t·ha^−1^ compared to the control (C). In particular, the yield performance and grain quality expressed the positive impact of treatments and the intrinsic genetic potential of the investigated varieties, since the *p*-value of their interaction (treatment × variety) was ≤0.01. In particular, the T2V2 (combined treatment × Moscovckaya 40 variety) combination recorded better results for all the variables studied, namely, grain yield, protein content, and disease resistance. Therefore, the Moscovckaya 40 variety (V2) seems to be a better performer than the Nemchinovckaya 17 variety (V1), given the recorded values of grain yield, protein content, and disease resistance. These results are similar to those of other conducted studies. Polityko et al. (2016) [62] demonstrated that the Moscovckaya 40 variety was the most resistant variety to fungal diseases among six studied varieties. More recently, in 2021, Sandokhandze et al. [63] reported that the Moskovskaya 40 variety, followed by Nemchinovka 17, had high adaptability to the environment of the Central Non-Black Earth region, giving high yield and grain quality.

Most of the research in the literature focused on achieving high yields and grain quality of wheat without considering economic profitability. Economic efficiency (EE) is the degree or the ability of a farmer to produce a given output level at the least cost [64]. EE has been grouped into the appropriate choice of input combination and the appropriate options for production function among all those activities in use by farmers [65]. In our study, the combined treatment (T2) resulted in the highest winter wheat yield, with a cost/benefit ratio of 2.38 payback; the cost/benefit ratio of biological treatment was 2.03 payback, and that of chemical treatment was 2.65 payback. Indeed, these results showed that the eco-friendly farming method can be used in wheat cropping to guarantee high yield, high profitability, and a healthy product.

## 4. Material and Methods

The objective of this study was to investigate the response of two Russian winter wheat varieties to the crop protection treatments in an integrated crop management system, understand the influence of these treatments on yield, yield components, grain quality, and disease control, and evaluate the economic efficiency of each treatment.

### 4.1. Plant Material

In this experiment, two varieties of winter wheat (*Triticum aestivum* L.) from Russia were studied: the Nemchinovckaya 17 variety (V1) and Moscovckaya 40 variety (V2).

The Nemchinovckaya 17 variety (V1) is characterized by short stems, early heading maturity, winter hardiness (overwintering at the level of 95–97%), and resistance to lodging and diseases (not affected by brown rust). The grain is large (1000-grain weight: 47–53 g). It has good quality indicators: protein content of 14.5% and gluten content in flour of >38%.

The Moscovckaya 40 variety (V2) is characterized by short stems, early heading maturity, winter hardiness, very good straw strength, and excellent resistance to lodging and a number of dangerous diseases such as brown rust, powdery mildew and loose smut. The grain is large (1000-grain weight: 45–48 g). The grain quality is high: protein content of 15.0% and raw gluten content in flour of 33.7%. It was included in the State Register of Wheat in 2008.

### 4.2. Site Description and Soil Characteristics

The field investigation for this study was conducted at the Moscow Research Institute of Agriculture “Nemchinovka” Odintsovo district, Russia (55°45′ N, 37°37′ E, and 200 m altitude). This experiment was performed during four consecutive growing seasons (2016–2017, 2017–2018, 2018–2019, and 2019–2020). The climate can be described as mid-continental with a mean annual rainfall of 712 mm (56% in the spring–summer season and 26% in autumn). The mean annual temperature (2016–2020) was +4.20 °C. The average temperature of the warm season (May–August) was +14.40 °C; the average monthly temperature in January and July was −10.40 and +18 °C, respectively.

The mean time of air temperature period was nearly 215 days, while the average period with a temperature of more than +10 °C (vegetation season) was 215 days. The average temperature of the winter season (November–March) was −6.70 °C. The average precipitation rate from May to September was 344 mm, and the hydrothermal index was 1.2–1.3 in the Moscow Region.

The soil was the soddy–podzolic type, had a pH of 5.9, bulk density of 1.09 g·cm^−3^, humus of 1.9%, oxidizable N of 129.5 kg·ha^−1^, extractable P of 224 mg·kg^−1^, and extractable K of 139 mg·kg^−1^. The quantity of fertilizer used was taken into account depending on the soil characteristics.

### 4.3. Experimental Design and Treatments

During the four years of the study, randomized complete block design was used with four replications for each treatment. Three treatments (biological treatment (T1), combined treatment (T2), and chemical treatment (T3)) and one control (C) and two winter wheat varieties (Nemchinovckaya 17 variety (V1) and Moscovckaya 40 variety (V2)]) were examined. The area of the experimental field was 960 m^2^.

Table 5 shows the description of experimental inputs for all three treatments. The fertilization process in pre-sowing was conducted with top dressing at the tillering and earing stages. Planting was performed at the beginning of September with a planter (seeder SN 16 PM) at the rate of five million seeds per hectare. The crop rotation implemented in the experimental field was planting legume spring cereals and winter cereals. The precursor of the investigated crop in the study was peas. The tillage took place before each growing season after harvesting the precursor crop, with a ploughing depth of 20–22 cm. A modern combine harvester was used to harvest wheat around mid-August at ripening stage. The chemical treatment (T3) was used for comparison with the other studied treatments (T1 and T2).

### 4.4. Disease Incidence Estimation

The incidence of various diseases was assessed at tillering, elongation of stem, and earing stages in all treatments (C, T1, T2, and T3) and the two studied winter wheat varieties (V1 and V2). Ten plants within each plot were randomly selected. The percentage of diseases (snow mold, root rot, powdery mildew, and *Fusarium*) was estimated by interpreting visual symptoms of disease. Subsequently, through isolation and growing of fungi using appropriate artificial media, and then using the microscope, the pathogens were diagnosed on the basis of morphological characteristics, i.e., spore morphology, sporulation patterns, production, and characteristics of sporulating structures producing asexual and sexual spore forms, which was used for taxonomic classification of fungi [66].

### 4.5. Determination of Protein Content

Grain quality was analyzed on the basis of protein content in the different wheat varieties. Protein content was measured for the two studied winter wheat varieties according to the applied treatment. The percentage of protein content was determined by calculating the total nitrogen concentration in grain using the Kjeldahl method [67]; then, Equation (1) was used [68].

Protein (%) = [(N × 100)/(100 − W)] × K,(1)

where N is the nitrogen content in the grain (%), W is the moisture content of the grain or its processed products (%), and K is conversion coefficient of nitrogen content to protein, equal to 5.7 for wheat.

### 4.6. Economic Efficiency Calculation

The economic efficiency (EE) was calculated for the three tested treatments (T1—biological, T2—combined, and T3—chemical) depending on the studied winter wheat varieties, using Equations (2)–(4) [69].

Gross income (RUB·ha^−1^) = Y × PW, (2)

where Y is the yield in kg·ha^−1^, and PW is the price of 1 kg of wheat (13 RUB).

Net income (RUB·ha^−1^) = GI − TC, (3)

where GI is the gross income, and TC is the treatment cost.

Cost–benefit ratio (payback) = NI/TC,(4)

where NI is the net income, and TC is the treatment cost.

### 4.7. Statiscal Analysis

Calculation of the mean and standard error of the mean (SEM), and statistical analysis were performed using statistical R software 4.2.0. The value of each variable was expressed as the mean ± SEM. The variables used for comparison purposes were the treatments according to the two tested varieties (Nemchinovckaya 17 variety (V1) and Moscovckaya 40 variety (V2)). Consequently, the differences between treatments were assessed using one-way ANOVA. However, the values were considered significant when *p* < 0.05. Linear regression was used to evaluate the relationship between the “number of productive stems” and “disease infestation” and between “yield” and the “number of productive stems”. A principal component analysis (PCA) was run on the correlation matrix of eight variables to extract relevant information from various datasets that were collected from this experimental study. PCA analysis was applied to better analyze the relationships among all studied variables (i.e., yield, yield components, protein content, and disease).

## 5. Conclusions

The current study suggests two eco-friendly crop protection treatments in an integrated winter wheat-crop management system under field conditions. The conducted experiments took into account the tillage system, the crop rotation, the fertilization system, the combination of different bioagents with chemical molecules, and the economic efficiency of treatments. These suggestions allowed (1) an efficient disease management of 1.8–2.2% (SM), 1.2–1.8 (RR), 0.9–1.4 (PM), and 0.9–1.2% (Fus), (2) grain wheat yield of 5.5–6.8 t·ha^−1^, (3) grain protein content of 13.6–15.2%, and (4) profitability (payback) estimated at a cost–benefit ratio of 2.03–2.38.

The present study concludes that the best measures to limit disease damage in winter wheat are regulation of the nutrition of the plants, crop rotation, chemical and biological seed treatment, and the introduction of resistant varieties. Furthermore, despite the affordability of the chemical treatment, the present study recommended using the biological or combined treatments for higher yield and grain quality, as well as healthy products.

Lastly, by improving the other modern techniques of winter wheat crop management in an integrated way (for instance, cultivar selection, crop protection, crop rotation, tillage system, and crop nutrition) and by analyzing the relationship between them, we can increase our wheat yields in a context of sustainable agriculture.

## Figures and Tables

**Table 1:** Diseases rates variation in each durum wheat variety under different studied treatments (2017–2020).

Treatments	Varieties	(SM)	(RR)	(PM)	(Fus)
Control	Nemchinovskaya 17 variety (V1)	31.7 ± 1.31 ^a^	16.8 ± 0.71 ^b^	12.2 ± 0.15 ^a^	10.7 ± 0.61 ^b^
	Moscovskaya 40 variety (V2)	29.6 ± 0.33 ^b^	17.8 ± 0.24 ^a^	11.9 ± 0.13 ^b^	11.9 ± 0.21 ^a^
Biological treatment (T1)	Nemchinovskaya 17 variety (V1)	2.0 ± 0.09 ^e^	1.8 ±0.14 ^cd^	1.3 ± 0.05 ^cd^	1.2 ± 0.06 ^c^
	Moscovskaya 40 variety (V2)	2.2 ± 0.14 ^de^	1.9 ± 0.10 ^c^	1.4 ± 0.09 ^cd^	1.2 ± 0.06 ^c^
Combined treatment (T2)	Nemchinovskaya 17 variety (V1)	1.8 ± 0.07 ^e^	1.2 ± 0.06 ^e^	0.9 ± 0.06 ^e^	0.9 ± 0.06 ^c^
	Moscovskaya 40 variety (V2)	1.8 ± 0.08 ^e^	1.6 ± 0.10 ^d^	1.0 ± 0.03 ^e^	0.9 ± 0.07 ^c^
Chemical treatment (T3)	Nemchinovskaya 17 variety (V1)	2.8 ± 0.17 ^c^	2.1 ± 0.07 ^c^	1.6 ± 0.16 ^c^	1.3 ± 0.09 ^c^
	Moscovskaya 40 variety (V2)	2.5 ± 0.17 ^cd^	2.1 ± 0.09 ^c^	1.3 ± 0.06 ^d^	1.2 ± 0.09 ^c^
*p*-Value	Treatment	≤0.001	≤0.001	≤0.001	≤0.001
	Variety	≤0.001	≤0.001	0.0570	0.0957
	Treatment × variety	≤0.001	≤0.001	≤0.01	≤0.05

Means followed by different letters are significantly different according to the Newman–Keuls LSA test (*p* ≤ 0.05).

**Table 2:** Grain yield and protein variation of each wheat varieties under different studied treatments (2017–2020).

Treatments	Varieties	Yield (t·ha^−1^)	Protein (%)
Control	Nemchinovskaya 17 variety (V1)	4.3 ± 0.17 ^e^	13.9 ± 0.57 ^c^
	Moscovskaya 40 variety (V2)	4.4 ± 0.18 ^e^	13.3 ± 0.04 ^c^
Biological treatment (T1)	Nemchinovskaya 17 variety (V1)	5.5 ± 0.04 ^cd^	13.6 ± 0.04 ^c^
	Moscovskaya 40 variety (V2)	5.5 ± 0.06 ^d^	13.7 ± 0.04 ^c^
Combined treatment (T2)	Nemchinovskaya 17 variety (V1)	6.3 ± 0.04 ^b^	15.1 ± 0.04 ^a^
	Moscovskaya 40 variety (V2)	6.8 ± 0.05 ^a^	15.2 ± 0.02 ^a^
Chemical treatment (T3)	Nemchinovskaya 17 variety (V1)	5.8 ± 0.03 ^c^	14.4 ± 0.06 ^b^
	Moscovskaya 40 variety (V2)	6.3 ± 0.08 ^b^	14.7 ± 0.03 ^b^
*p*-Value	Treatment	≤0.001	≤0.001
	Variety	≤0.001	0.876
	Treatment × Variety	≤0.01	0.157

Means followed by different letters are significantly different according to the Newman–Keuls LSA test (*p* ≤ 0.05).

**Table 3:** Yield components of each wheat variety under different studied treatments (2017–2020).

Treatments	Varieties	NPS (stems/m^2^)	1000-Grain Weight (g)
Control	Nemchinovskaya 17 variety (V1)	537.6 ± 2.51 ^e^	37.3 ± 0.76 ^d^
	Moscovskaya 40 variety (V2)	522.2 ± 1.43 ^f^	41.5 ± 0.17 ^b^
Biological treatment (T1)	Nemchinovskaya 17 variety (V1)	552.5 ± 0.97 ^b^	38.1 ± 0.21 ^c^
	Moscovskaya 40 variety (V2)	548.8 ± 0.95 ^cd^	43.6 ± 0.24 ^a^
Combined treatment (T2)	Nemchinovskaya 17 variety (V1)	556.4 ± 1.77 ^a^	38.4 ± 0.15 ^c^
	Moscovskaya 40 variety (V2)	551.8 ± 0.84 ^bc^	43.7 ± 0.27 ^a^
Chemical treatment (T3)	Nemchinovskaya 17 variety (V1)	551.7 ± 0.99 ^bc^	38.0 ± 0.29 ^c^
	Moscovskaya 40 variety (V2)	548.3 ± 0.93 ^d^	43.4 ± 0.28 ^a^
*p*-Value	Treatment	≤0.001	≤0.001
	Variety	≤0.001	≤0.001
	Treatment × Variety	≤0.001	≤0.05

Means followed by different letters are significantly different according to the Newman–Keuls LSA test (*p* ≤ 0.05).

**Table 4:** Economic efficiency of two winter wheat varieties as affected by treatments (average for 2017–2020).

Varieties	Treatments	Yield, (t·ha^−^^1^)	Gross Income, (RUB·ha^−^^1^)	Treatments Cost, (RUB·ha^−^^1^)	Net Income (RUB·ha^−^^1^)	Cost–Benefit Ratio (Payback)
Nemchinovskaya 17 variety (V1)	T1	5.5	71,500	23,550	47,950	2.03
	T2	6.3	81,900	26,100	55,800	2.13
	T3	5.8	75,400	22,400	53,000	2.36
Moscovskaya 40 variety (V2)	T1	5.5	71,500	23,550	47,950	2.03
	T2	6.8	88,400	26,100	62,300	2.38
	T3	6.3	81,900	22,400	59,500	2.65

**Table 5:** Applied treatments in the crop protection system.

Treatments	Fertilizers (kg·ha^−1^)	Crop Protection Details
1. Control	Basal application N (60), P_2_O_5_ (90), K_2_O (120) (kg/ha) in pre-sowing, top dressing, at the tillering and earing phases, N (30) and N (30) (kg/ha), respectively	-
2. Biological treatment (T1)		SPOREX—2.0 L/t (*Bacillus subtilis + Bacillus megaterium)*: pre-sowing seed treatment; ALIRIN-B—2.0 L/ha (*Bacillus subtilis*) + PLANRIZOM—2.0 L/ha (*Pseudomonas fluorescens*) + GLIOCLADIN—2.0 L/ha. (*Trichoderma harzianumat*) at tillering–elongation of stem phases; GAMAIR—3.0 L/ha (*Bacillus subtilis*) + GLIOCLADIN—3.0 L/ha (*Trichoderma harzianumat*) at earing–flowering phases.
3. Combined treatment (T2)		SPOREX—1.0 L/t (*Bacillus subtilis + Bacillus megaterium*) + VINCIT FORTE—0.75 L/t (active molecules: flutriafol + thiabendazole + imazalil): pre-sowing seed treatment; ALIRIN-B—1.0 L/ha (*Bacillus subtilis*) + PLANRIZOM—1.0 L/ha (*Pseudomonas fluorescens*) + GLIOCLADIN—1.5 L/ha. (*Trichoderma harzianumat*) + SUPER IMPACT—0.5 L/ha (active molecules: flutriafol + tebuconazole) at tillering and elongation of stem phases; PLANRIZOM—1.0 L/ha (*Pseudomonas fluorescens*) + GLIOCLADIN—1.0 L/ha (*Trichoderma harzianumat*) + CONSUL 1 L/ha (active molecules: flutriafol + azoxystrobine) + GAMAIR—1.0 L/ha (*Bacillus subtilis*) at earing phase.
4. Chemical treatment (T3)		Fungicides: VINCIT FORTE—1.25 L/t (active molecules: flutriafol + thiabendazole + imazalil): pre-sowing seed treatment; SAPRESS—0.3 L/h (active molecules: trinexapac-ethyl) at tillering phase; SUPER IMPACT—0.75 L/ha (active molecules: flutriafol + tebuconazole) + SAPRESS—0.3 L/h (active molecules: trinexapac-ethyl) at elongation of stem phase; CONSUL—1.0 L/ ha (active molecules: flutriafol + azoxystrobine) at earing phase.

## Data Availability

Not applicable.
